# Identification of a 14-Gene Prognostic Signature for Diffuse Large B Cell Lymphoma (DLBCL)

**DOI:** 10.3389/fgene.2021.625414

**Published:** 2021-02-10

**Authors:** Pengcheng Feng, Hongxia Li, Jinhong Pei, Yan Huang, Guixia Li

**Affiliations:** ^1^Department of Basic Medicine, Changzhi Medical College, Changzhi, China; ^2^Affiliated Hospital of Changzhi Institute of Traditional Chinese Medicine, Changzhi, China

**Keywords:** diffuse large B cell lymphoma, immune-related gene, immune prognostic model, risk score formula, immune infiltration

## Abstract

Although immunotherapy is a potential strategy to resist cancers, due to the inadequate acknowledge, this treatment is not always effective for diffuse large B cell lymphoma (DLBCL) patients. Based on the current situation, it is critical to systematically investigate the immune pattern. According to the result of univariate and multivariate cox proportional hazards, LASSO regression and Kaplan-Meier survival analysis on immune-related genes (IRGs), a prognostic signature, containing 14 IRGs (AQP9, LMBR1L, FGF20, TANK, CRP, ORM1, JAK1, BACH2, MTCP1, IFITM1, TNFSF10, FGF12, RFX5, and LAP3), was built. This model was validated by external data, and performed well. DLBCL patients were divided into low- and high-risk groups, according to risk scores from risk formula. The results of CIBERSORT showed that different immune status and infiltration pattern were observed in these two groups. Gene set enrichment analysis (GSEA) indicated 12 signaling pathways were significantly enriched in the high-risk group, such as natural killer cell-mediated cytotoxicity, toll-like receptor signaling pathway, and so on. In summary, 14 clinically significant IRGs were screened to build a risk score formula. This formula was an accurate tool to provide a certain basis for the treatment of DLBCL patients.

## Introduction

Diffuse large B cell lymphoma (DLBCL) is the most common subtype of non-hodgkin lymphoma (NHL), it can be divided into three molecular subtypes [germinal center B cell (GCB) subtype, activated B cell (ABC)-like subtype, and the unclassified subtypes.] according to the unique genetic signatures (Calado et al., 2010; Zhang et al., 2016). It has been thought as an aggressive disease caused by rapidly dividing malignant B cells. With further research of deeper genome sequencing and transcriptomic profiling, it has been proven that the complexity of DLBCL biology was seriously underestimated (Scott and Gascoyne, 2014; Opinto et al., 2020).

The majority of DLBCL patients could be relieved after a standard regimen of rituximab in combination with chemotherapy, however, 40% of DLBCL patients had a poor prognosis without suitable curative therapies (Kim et al., 2012; Coiffier and Sarkozy, 2016; Carpìo et al., 2020; Zhou et al., 2020). Based on this situation, the researches of the treatment strategies on DLBCL remain important.

One of the features in the occurrence and development of carcinoma is the change of immune status. Tumor immune evading mechanisms were increasingly recognized crucial in the formation and development of multiple cancer (Motzer et al., 2014; Velcheti et al., 2014; Borghaei et al., 2015; Lin et al., 2016; Wallin et al., 2016). The fact decreased immunity stimulated the growth of cancer cells could be reversed, with the emergence of immunotherapy (Silver et al., 2015). Hence, cancer immunotherapy has become one of the major strategies to treat cancer and the researches about the relationship between immune cell and tumor have become a hot topic (Schumacher and Schreiber, 2015; Liu et al., 2017; Popovic et al., 2018; Sebastian et al., 2018). It is generally believed that a single immune marker is too farfetched to illustrate the complex immune environment. Therefore, it is necessary to find a multi-immune relevant-gene-based signature to help the physician predict patients’ prognosis and characteristic of tumor microenvironment.

The therapy of immune checkpoint blockades had achieved unprecedented success in helping many cancer patients to extend overall survival (OS) (Gettinger et al., 2016; Reck et al., 2016; Rittmeyer et al., 2016). So, in the process of curing cancer, immunotherapy is always an important consideration. However, the benefited population was limited due to high heterogeneity in biological and clinical appearances (Georg et al., 2010; Dobashi and Akito, 2016; Gentzler et al., 2016). Several immune checkpoint inhibitors could enhance cytotoxicity by targeting programmed cell death protein 1 (PD-1) (CD279), programmed cell death ligand 1 (PD-L1) (CD274), cytotoxic T lymphocyte antigen-4 (CTLA-4), lymphocyte activation gene 3 (LAG-3) (CD223), and T cell immunoglobulin-3 (TIM-3) (HAVCR2). PD-1/PD-L1 could cause the host immune evasion and promotion of metastasis (Velcheti et al., 2014). CTLA-4 belonged to immunoglobulin- related receptors family and could respond to T-cell immune negative regulation (Rowshanravan et al., 2017; Hosseini et al., 2020). Blocking the expression of PD-1 and CTLA-4 improved the outcomes of patients in different cancers, but immune-related adverse events were observed. LAG-3, an immune inhibitory receptor, was regarded as the foremost target next to PD-1.

In this work, we combined clinical information with immune-related genes (IRGs) expression profiles from 216 DLBCL patients to evaluate the OS. The risk score formula was constructed to predict the individual survival time. Furthermore, the prognosis significance of multiple immune biomarkers was confirmed by the cancer genome atlas (TCGA)-DLBC and GSE32918. This result provided a model for immune-related work and was the critical step toward developing personalized strategies for DLBCL.

## Materials and Methods

### Data Collection

The level-3 RNA-seq data and clinical data of DLBCL were downloaded from the TCGA and normalized by TCGAbiolinks R package. The raw datasets (GSE136971 and GSE32918) of DLBCL were downloaded from the gene expression omnibus (GEO) database. Limma package was used to screen the differential expression genes. Perl was used to transform ensemble IDs and probe names to symbols, separately. IRGs were obtained from the Immunology Database and Analysis Portal (ImmPort)^1^. Univariate cox proportional hazard regression was used to associate the IRGs with DLBCL patients’ OS. Only IRGs with *P* value less than 0.05 were selected as putative genes for further analysis. Least absolute shrinkage and selection operator (LASSO) regression was performed to prevent the model overfitting, using ten-fold cross-validation to exam penalty parameter. Multivariate cox regression analysis was performed to assess the risk value of each IRGs signature, then a risk score was established as following:

Risk score = β gene1 × gene1 expression value + β gene 2 × gene 2 expression value + β gene 3 × gene 3 expression value + ⋯⋯ + β gene n × gene n expression value. n is the number of relative IRGs, β is the coefficient generated by the multivariate cox regression.

All data were downloaded from public databases and did not apply for approval of the local ethics committees. A methodological flowchart of this research was shown in Supplementary Figure 1.

### IRGs Signature Construction and Confirmation

Low- and high-risk groups were generated based on the median risk scores of DLBCL patients. Kaplan-Meier (K-M) was performed to estimate survival distribution. “TimeROC” and “survival” packages were used to examine the suitability of survival prediction among risk models.

### Tumor-Infiltrating Immune Cells

CIBERSORT from sangerbox^2^ was used to explore the abundance of tumor-infiltrating immune cells. Ninety-eight IRGs were submitted to CIBERSORT, to predict the roles of immune infiltration in DLBCL. The correlations between IRGs and four immune checkpoints were analyzed using TCGA-DLBC tumor data by GEPIA^3^. The significant level was less than 0.05.

### GSEA-Enrichment Analysis

To explore the potential biological function of IRGs, gene set enrichment analysis (GSEA) (v 4.1.0) was carried out, based on the gene expression data from low- and high-risk groups. C2.cp.KEGG.v7.2. symbols. gmt was selected as reference gene set database. Enrichment pathways were filtered under the condition of *P* value less than 0.05 and FDR *P* value less than 0.25.

### CMap Analysis

Connectivity Map (CMap) (version 02)^4^, was adopted to screen putative drugs targeting 14 IRGs. CMap is a website used to search connections among genes, diseases and drugs. All probe IDs, corresponding to 14 IRGs on HG-U133A, were obtained according to GPL96. The genes that hazard ratios were greater than 1 were marked “up” and less than were marked “down.” The probe ID was input into files with “up” and “down” tags saved as “.grp” format. Small molecular drugs that were negatively correlated with the 14 IRGs signature might have the potential to treat DLBCL.

### The Analysis of 14 IRGs Expression Level

The expression matrixes were searched in GEO database using lymph as a keyword to explore the expression level of 14 IRGs. The samples (GSM217767, GSM217768, GSM217769, GSM217770, GSM217771, GSM217772, GSM217773, GSM217774, and GSM217775) in GSE8762 were used as control. GSE64555 and GSE159472 were used as disease data sets. These three data were annotated with GPL570. In order to reduce the differences caused by different standardization methods in GEO data, two R packages, Affy and affPLM, were used to re-standardize the original data.

## Results

### The Preparation and Description of Clinical Data and Expression Data

Only individuals with complete clinical information could be used as experimental samples. In order to reduce errors as much as possible and make our model more reliable, the subjects which the survival time were less than 100 days and no survival information were abandoned. GSE136971, containing 216 samples, were used as a training group. No survival status information was recorded for GSM2329007 and GSM2329133. The survival time of GSM2329094, GSM2329071, GSM2329069, GSM2329022, and GSM2329976 was less than 100 days. These seven individual samples were deleted. GSE32918 (189 samples) and TCGA-DLBC (44 samples) were used as a validating group.

### A Risk Formula Was Constructed Using Fourteen-Four IRGs

**FIGURE 1:**
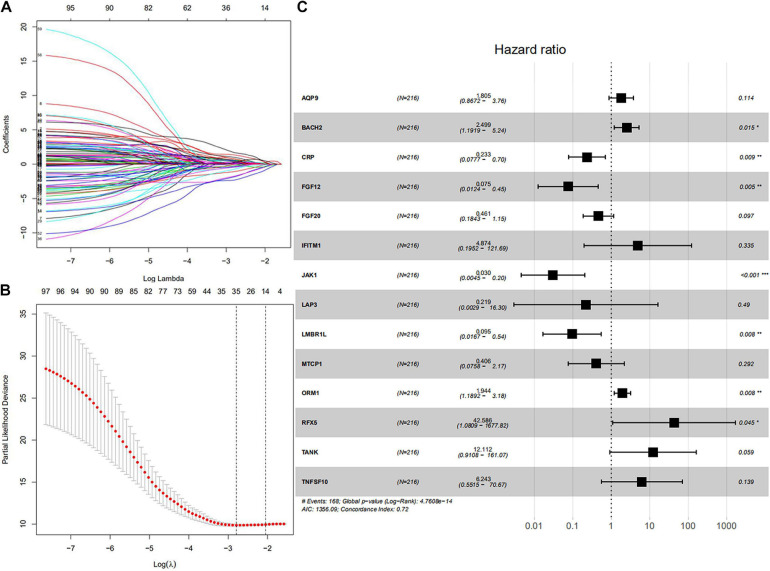
Core IRGs OS relevant were Identified by Cox analysis. **(A)** LASSO coefficient profiles for 98 significant IRGs in univariate Cox. **(B)** Cross-validation for selecting the tuning parameters for the LASSO model. **(C)** Forest plots showed the relationships of 14 IRGs with OS in the training group. The unadjusted hazard ratios are presented with 95% CIs. ^∗^*P* < 0.05, ^∗∗^*P* < 0.01, and ^∗∗∗^*P* < 0.001.

All symbol and synonyms of IRGs from ImmPort were downloaded, to avoid omissions. 7887 IRGs were obtained and summarized in Supplementary Table 1, 1328 IRGs were screened by merging the expression data of 7887 IRGs and GSE136971. Ninety-eight IRGs were related to OS and screened by univariate Cox proportional hazard regression. The detailed results of univariate Cox proportional hazard regression were shown in Supplementary Table 1. Fourteen IRGs (AQP9, LMBR1L, FGF20, TANK, CRP, ORM1, JAK1, BACH1, MTCP1, IFITM1, TNFSF10, FGF12, RFX5, and LAP3) were identified by LASSO regression (Figures 1A,B). These IRGs were used to predict risk score by multivariate cox regression (Figure 1C). According to the risk coefficient of 14 IRGs from multivariate cox regression, a risk score formula was constructed as follows.

Risk score = (expression of AQP9 × 1.80452) + (expression of BACH2 × 2.49894) + (expression of CRP × 0.2329) + (expression of FGF12 × 0.07525) + (expression of FGF20 × 0.46076) + (expression of IFITM1 × 4.874) + (expression of JAK1 × 0.03042) + (expression of LAP3 × 0.2187) + (expression of LMBR1L × 0.09529) + (expression of MTCP1 × 0.40574) + (expression of ORM1 × 1.94444) + (expression of RFX5 × 42.58626) + (expression of TANK × 12.11217) + (expression of TNFSF10 × 6.24265). The result of multivariate Cox regression was shown in Table 1.

**TABLE 1:** The risk coefficient of 14 IRGs.

ID	exp(coef)	exp(-coef)	Low 95% CI	Low 95% CI	*P* value
AQP9	1.80452	0.55416	0.867163	3.7551	0.11439
BACH2	2.49894	0.40017	1.191857	5.2395	0.015325
CRP	0.2329	4.29369	0.077665	0.6984	0.009307
FGF12	0.07525	13.28981	0.012446	0.4549	0.004833
FGF20	0.46076	2.17031	0.18434	1.1517	0.097356
IFITM1	4.874	0.20517	0.195216	121.6904	0.334628
JAK1	0.03042	32.86862	0.004527	0.2045	0.000327
LAP3	0.2187	4.57247	0.002935	16.2981	0.489525
LMBR1L	0.09529	10.49381	0.016692	0.544	0.008172
MTCP1	0.40574	2.46462	0.075827	2.1711	0.29185
ORM1	1.94444	0.51429	1.189246	3.1792	0.008028
RFX5	42.58626	0.02348	1.080917	1677.8247	0.045341
TANK	12.11217	0.08256	0.910807	161.0709	0.058865
TNFSF10	6.24265	0.16019	0.551469	70.667	0.139076

In this part, the relationship between gender and OS of DLBCL patients were explored, but there was no significant correlation between gender and survival.

### Using 14 IRGs Construct the Prognostic Risk Signature for DLBCL

**FIGURE 2:**
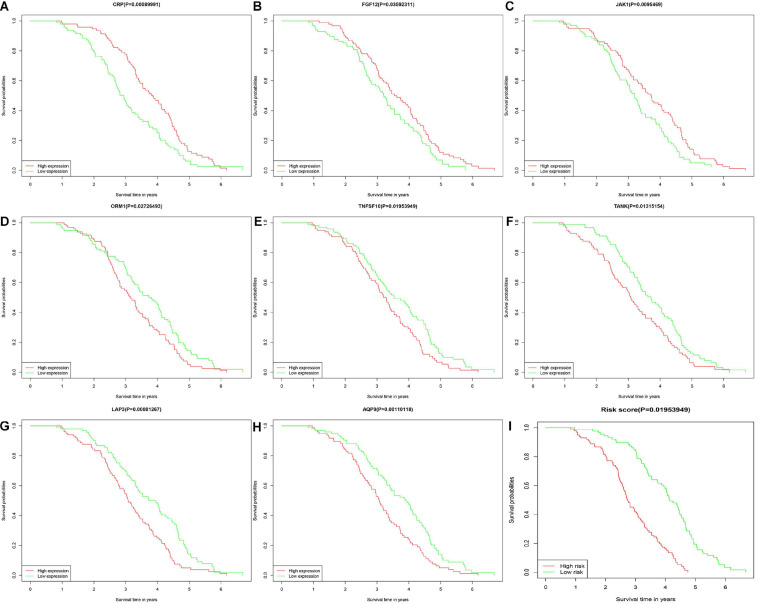
Kaplan-Meier plotters showed about overall survival of 14 IRGs in DLBCL. The horizontal axis represented the survival time by month or year. The vertical axis represented survival probability. The high-risk group marked red and low-risk group marked green. There were six IRGs with no difference in OS between high and low risk groups. **(A–C)** CRP, FGF12, and JAK1. **(D–H)** ORM1, TNFSF10, TANK, LAP3, and AQP9. **(I)** The low-risk group and high-risk group.

**FIGURE 3:**
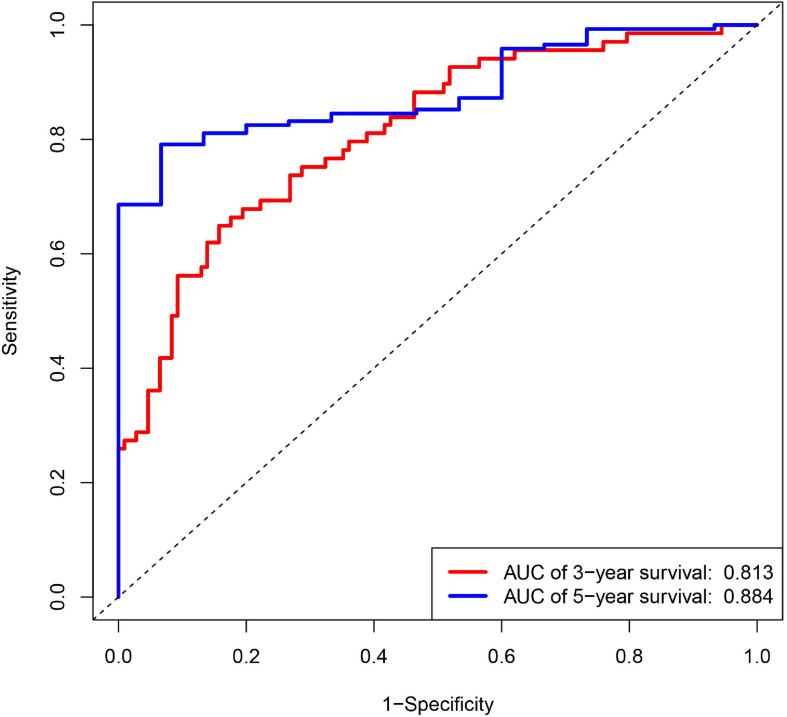
Receiver operating characteristic (ROC) curves for 3- and 5-year survival probability according to 14 IRGs signature in the training group.

Diffuse large B cell lymphoma patients were divided into low- and high-risk groups, according to the risk score calculated by formula, the median of the risk value was served as the cutoff value (cutoff = 1682). Survival curve and ROC curve were performed to test the suitability of the module. As shown in Figures 2A–H, the high expression of JAK1, CRP, and FGF12, may increase the risk of death, while, the high expression of AQP9, LAP3, ORM1, TANK, and TNFSF10, may increase the chance of survival. The K-M curve also indicated worse prognosis in the high-risk groups (Figure 2I). The areas under the curve (AUC) of 3- and 5-year ROC curve for the prognosis model were 0.813 and 0.884 (Figure 3).

### Verification of the Prognostic Value of 14 IRGs Biomarkers

**FIGURE 4:**
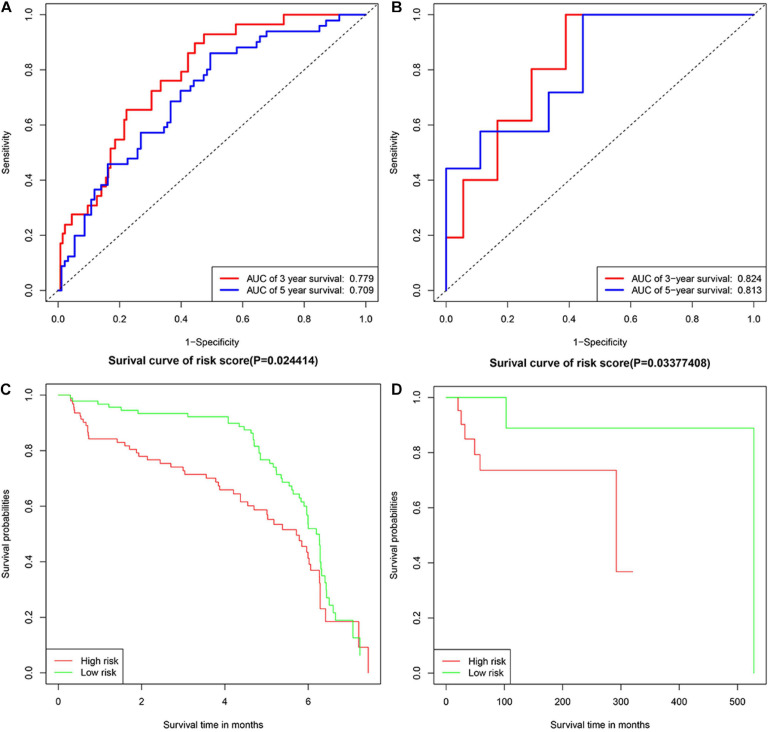
The Kaplan-Meier plotters and ROC curves in validation groups. Panels **(A,B)** represented Kaplan-Meier plotters in TCGA-DLBC and GSE32918, separately. Panels **(C,D)** represented ROC curves in TCGA-DLBC and GSE32918, separately.

GSE32918 and TCGA-DLBC were used as validation cohorts in this work. Samples were divided into two groups according to the median risk score. Verification results were consistent with expectations, with the risk score increasing, the number of deaths increased. The AUC for 3- and 5-years survival in validation groups of GSE32918 were 0.779 and 0.709. The AUC for 3- and 5-years survival in validation groups of TCGA-DLBC were 0.824 and 0.813. The result of the K-M survival curve and ROC were shown in Figure 4.

### Functional Annotation of the IRGs

**FIGURE 5:**
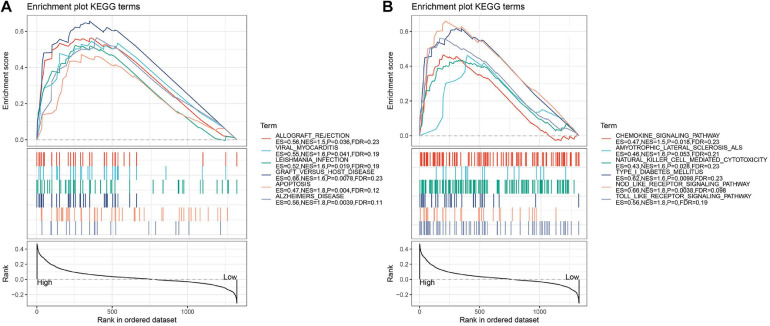
**(A,B)** GSEA pathways enriched in the low- and high-risk groups according to the immune-related genes. There are too many results of GSEA enrichment, so it is divided into two graphs, panels **(A,B)**. Pathways enriched in the low-risk group. Vertical lines represented the positions of genes belonging to the set of special pathways. Negative enrichment score indicates a higher correlation with individuals in the low-risk group.

Based on the GSE136971 expression data, we explored the difference between low- and high-risk groups using GSEA. Several significant enrichment signaling pathways were detected. Twelve significant pathways were differentially enriched in the low and high-risk groups, including chemokine signaling pathway, allograft rejection, viral myocarditis, leishmania infection, natural killer cell-mediated cytotoxicity, type I diabetes mellitus, graft versus host disease, amyotrophic lateral sclerosis (ALS), nod like receptor signaling pathway, apoptosis, Alzheimers’ disease and toll-like receptor signaling pathway (Figure 5 and Table 2).

**TABLE 2:** Detailed information of KEGG from GSEA.

KEGG NAMES	ES	NES	NOM p	FDR q
Toll like receptor signaling pathway	0.561	1.830	0.000	0.186
Alzheimers disease	0.565	1.811	0.004	0.114
apoptosis	0.472	1.763	0.004	0.123
Nod like receptor signaling pathway	0.659	1.757	0.004	0.098
Type I diabetes mellitus	0.617	1.637	0.010	0.233
Graft versus host disease	0.657	1.616	0.008	0.230
Natural killer cell mediated cytotoxicity	0.434	1.598	0.028	0.229
Amyotrophic lateral sclerosis (ALS)	0.463	1.594	0.053	0.207
Leishmania infection	0.522	1.589	0.019	0.190
Viral myocarditis	0.545	1.575	0.041	0.192
Allograft rejection	0.564	1.539	0.036	0.227
Chemokine signaling pathway	0.465	1.523	0.018	0.232

### Correlation Analysis Between IRGs and Immune Checkpoints

**FIGURE 6:**
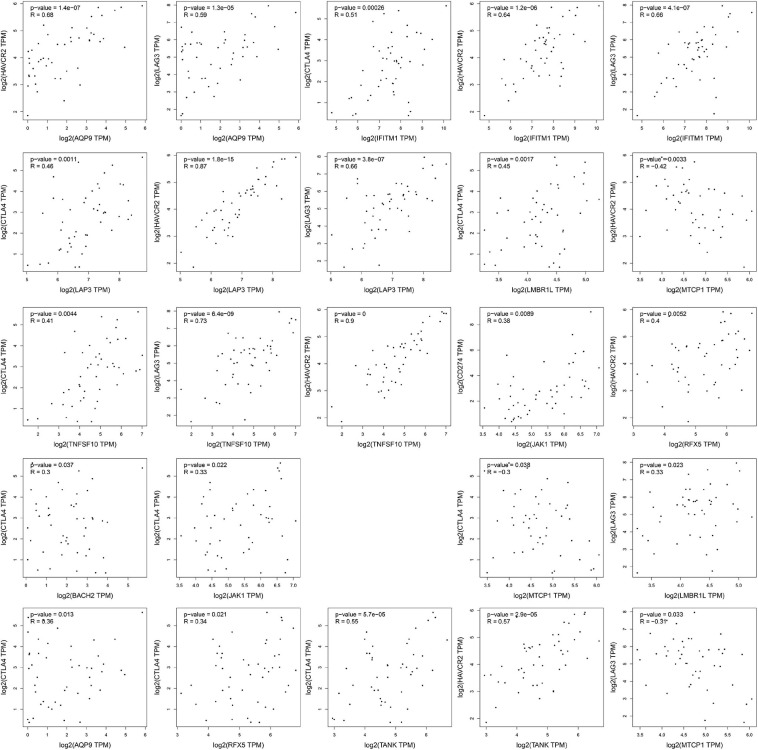
The results of correlation analysis. The expression data of all gene had taken the logarithmic value of 2. The statistically insignificant experimental results were not shown.

The four important immune checkpoints (LAG3, TIM3, CTLA-4, and PD-1/PD-L1) were widely used in cancer immunotherapy. To investigate the possible role of fourteen IRGs in ICB (immune checkpoint Blockade) therapy, the association of fourteen IRGs and four immune checkpoints were analyzed by Pearson’s correlation analysis. Only the gene pairs which the *P*-value was less than 0.05 were shown in Figure 6. MTCP1 was negatively related to HAVCR2 (−0.42, *P* = 0.0033), TIM3 (*R* = −0.31, *P* = 0.033), and CTLA4 (*R* = −0.3, 0.038). The other 21 gene pairs were positive which *R* values were from 0.38 to 0.9, *P* values were less than 0.05.

### Immunocyte Infiltration in the Microenvironment

**FIGURE 7:**
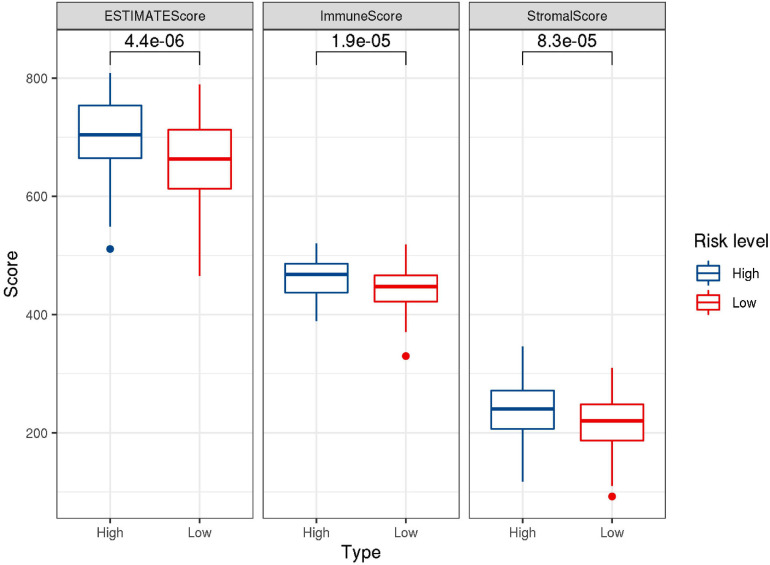
Box-plots showed the difference in the scores of ESTIMATE, immune and stromal between the low- and high-risk groups.

**FIGURE 8:**
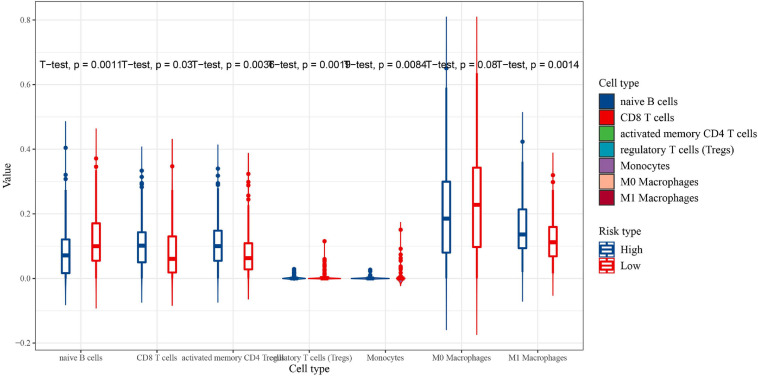
The proportion of immune cell infiltration between the low-risk group (red) and high-risk group (blue) (significant differences between high and low risk groups).

CIBERSORT was performed to understand the connection between IRGs and immune cell infiltration. The proportion of 22 immune cells was estimated according to the expression data of GSE136971. The immune score, stromal score and ESTIMATE score were calculated by ESTIMATE algorithm (Figure 7). Low-risk groups had higher level of immune infiltration. Furthermore, a significant difference was observed for the immune score (*P* = 1.9e-5) and ESTIMATE score (*P* = 4.4e-6). However, the difference of stromal score between low- and high-risk groups was not significant (*P*≈0.056). Six immune cells were observed between low- and high-risk groups (Figure 8). The higher expression level of CD8 T cells, CD4 memory T cells activated and M1 macrophages were shown in low-risk samples. Naïve B cells, regulatory T cells (Tregs) and monocytes were higher in high-risk individuals. In other 16 immune-related cells, expression differences were not statistically significant (Supplementary Figure 2).

### Small Molecular Drugs Predicted by CMap

More than one probe in HG-U133A array was found to correspond to 14 IRGs, 15 probes were input “up” file and nine were input “down” file. Fourteen IRGs were uploaded to CMap to identify compounds that cured DLBCL, and ranked based on enrichment score (from −0.976 to 0.979) to screen the top 79 small molecular compounds (*P* ≤ 0.05) (Supplementary Table 2). The drugs without *P* values were excluded. Therefore, these drugs might be the most promising novel candidates for DLBCL treatment.

### Detect the Expression Level of 14 IRGs

**FIGURE 9:**
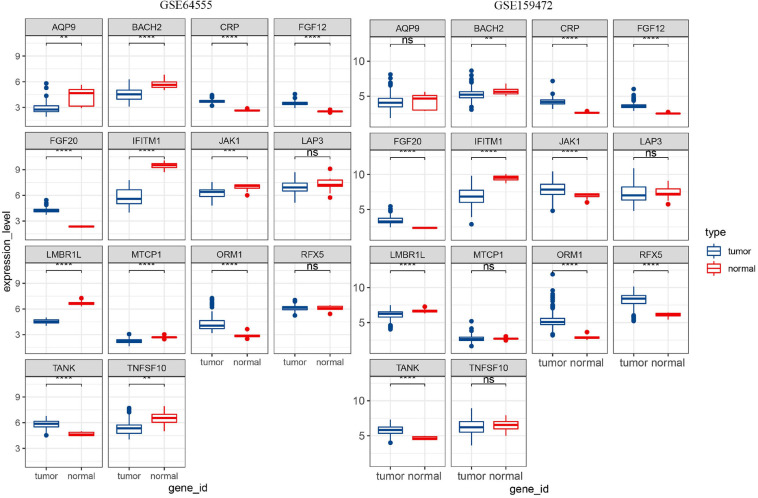
The results of differential expression of 14 IRGs in two GEO data sets were shown.

The expression data of 14 IRGs were extracted from GSE8762, GSE64555, and GSE159472. The results of the differential expression of the 14 IRGs in the three data sets were same, except for JAk1. The differential expressions of JAK1 were significant higher expressed in disease samples both GSE136971 and GSE159472, however, in GSE64555, the expression of the normal samples was higher and in GSE159472 disease samples higher (Figure 9). It is possible that in the process of selecting test populations, differences in human bodies in different regions, or differences in some test populations, resulted in completely opposite results in the same disease sequencing process.

## Discussion

Although the combination treatment of Rituximab and standard CHOP chemotherapy had achieved unprecedented success in the prognosis and cure of DLBCL patients. However, the treatment of DLBCL is still tricky. Recently, immunotherapy is considered as a most potential treatment strategy and has shown strong strength in the treatment of cancers (Pitt et al., 2016; Llovet et al., 2018). As the present single biomarkers were not reliable enough to predict benefit from ICB therapy, the beneficiary group in DLBCL patients was few. It is essential to construct a multi-immune relevant-gene-based signature and analyze the correlation between the IRGs genes and immune checkpoints (Mushtaq et al., 2018).

Numerous previous studies had shown multi-immune related genes (IRGs) could be used as diagnostic tools and provided advice for the physician in multiple cancers (She et al., 2020; Zhang et al., 2020). However, the potential role of IRGs was not clear in DLBCL. Thus, a prognostic model was developed, which fourteen genes were included (AQP9, LMBR1L, FGF20, TANK, CRP, ORM1, JAK1, BACH1, MTCP1, IFITM1, TNFSF10, FGF12, RFX5, and LAP3), at the same time, its value of prognostic and prediction were analyzed.

The same gene may have different functions in different diseases. Poor prognostic factors had similar roles in different cancers, such as stimulating the proliferation and metastasis of tumor cell. High expression levels of AQP9 in renal cell carcinoma individual had the trend of bad prognosis (Yamada et al., 2019). However, high expression levels of AQP9 in gastric cancer and colorectal cancer patients were correlated with better OS (Huang et al., 2017; Thapa et al., 2018). The detailed information of the role of 13 IRGs play in different disease was shown in Table 3. All in all, our analysis results were in agreement with previous researches about these 14 genes. Therefore, all the present genes in this paper could be predicted as candidates for prognostic markers of DLBCL.

**TABLE 3:** The information of 13 IRGs related diseases and functions.

IRGs	Disease type	Functions
AQP9	Astrocytoma (Lv et al., 2018), hepatocellular carcinoma (Liao et al., 2020), breast cancer (Zhu et al., 2019), renal cell carcinoma (Yamada et al., 2019), melanoma (Gao et al., 2011) colorectal cancer and gastric cancer (Huang et al., 2017; Thapa et al., 2018)	Promote the invasion and motility inhibit cell apoptosis Overexpression of AQP9 were correlated to have better OS
LMBR1L	In mice (Choi et al., 2019)	Mutant LMBR1L impaired the development of lymphoid lineages
FGF20	Human hepatocellular carcinoma cells (Wang et al., 2017)	Stimulate the proliferation and migration of cancer cells
TANK		Antiviral innate immunity and negatively regulates NF-NF-κB signaling pathway (Kadkhodazadeh et al., 2012; Song et al., 2012).
CRP	BLBCL (Cao et al., 2012; Wang et al., 2016), breast cancer, non-metastatic clear cell renal cell carcinoma, non-small cell lung cancer and colorectal cancer risk (Nimptsch et al., 2015; Hu et al., 2016; Akamine et al., 2018; Preet et al., 2018a, b)	Inflammatory marker
ORM1	Rat model of septic stroke pathology	Neuroinflammation (Astrup et al., 2019; Higuchi et al., 2020; Mcguckin et al., 2020; Sharma et al., 2020)
JAK1		Immune deficiency in natural killer cells (Witalisz et al., 2019), the mutation of JAK1 was related to immune escape in many cancers (Xie et al., 2009; Albacker et al., 2017) apoptosis and growth in several cancers (Siavash et al., 2004) optimal fitness of activated B cell (Zhu et al., 2017)
BACH1	Lung cancer (Wiel et al., 2019), epithelial ovarian cancer, colon cancer, prostate cancer and colorectal cancer (Davudian et al., 2016; Shajari et al., 2017; Zhu et al., 2018; Han et al., 2019)	Stimulates glycolysis dependent lung cancer metastasis required for metastatic AsPC-1 cells (Sato et al., 2020) stimulate cancer cell metastasis
IFITM1	Lung cancer, colorectal cancer, inflammatory breast cancer, cervical squamous cell carcinoma and lung cancer (FangYu et al., 2015; Ogony et al., 2016; Jin et al., 2017; Zheng et al., 2017; Yan et al., 2019)	Silenced IFITM1 caused inhibited migration and invasion
TNFSF10	Amyloid-related disorders (Cantarella et al., 2015)	Stimulated proliferation and inflammation, and inhibited apoptosis (Huang et al., 2019) improvement and restrained immune/inflammatory response
FGF12	Esophageal squamous cell carcinoma (Bhushan et al., 2017a)	Silencing FGF12 inhibited apoptosis of radiation-induced cell and the cell migration and proliferation (Fumiaki et al., 2008; Bhushan et al., 2017b)
RFX5	Hepatocellular carcinoma	Promoted the progression of the cell cycle (Chen et al., 2020)
LAP3	Glioma cells (He et al., 2015), esophageal squamous cell (Zhang et al., 2014), ovarian carcinoma (Suganuma et al., 2005; Wang et al., 2015) breast cancer (Wang et al., 2020) and hepatocellular carcinoma (Tian et al., 2014)	Regulate cell proliferation, invasion and/or angiogenesis expressed in several malignant and affects tumor angiogenesis

The impact of the sample size and external validation on the model were taken into account. GSE136971, containing 216 available individual samples, was used as a training group. Two external data were used to validate the validity of the model. DLBCL patients were divided into low- and high-risk score groups, risk score value was used as a parameter. The gene expression evaluating strategies of the different datasets might be different, especially, data was from different sources platforms. Different cutoff was used to eliminate the potential difference in training groups and validation groups. The high-risk groups tended to have worse OS; the prognostic value of this model remained robust in two validation groups. Moreover, the AUC values of the training groups and validation groups were larger than 0.7, so the model was reliable.

Gene set enrichment analysis was performed to have a deeper understand of the underlying molecular mechanisms of the occurrence and development of DLBCL. GSEA enrichment indicated that these pathways (chemokine signaling pathway, allograft rejection, viral myocarditis, leishmania infection, natural killer cell-mediated cytotoxicity, type I diabetes mellitus, graft versus host disease, nod like receptor signaling pathway, apoptosis, Alzheimer’s disease and toll-like receptor signaling pathway.) were significantly related to the development of DLBCL. The abnormal expression of some chemotaxis, such as CCL3 and CCL4, were associated with bad prognosis in DLBCL (Takahashi et al., 2015). Viral myocarditis pathway involved autoimmune diseases (Zheng et al., 2016). The high expression of possible poor prognostic biomarker GJB2 caused bad prognostic through natural killer cell-mediated cytotoxicity pathway (Tang et al., 2020). Intrinsic anti-apoptosis was related to drug resistance and eventual fatal outcome in DLBCL patients to some extent (Muris et al., 2007; Cillessen et al., 2010; Liu et al., 2021).

Surprisingly, many studies had proved that there were gender differences in the occurrence and development of many diseases (Haitao et al., 2020; Strope et al., 2020). However, in this study, it was found that the OS rate and immune cell infiltration of DLBCL was not significantly related to gender. The differences in immune cells of gender in the same risk group were studied, but no significant differences were found. Male and female DLBCL patients may have the same immune pattern.

Our analysis result from ESTIMATE showed that high-risk group had higher immune cell infiltration scores. These research results were similar to the study of immune-related LncRNA in breast cancer (Shen et al., 2020). Naïve B cells and M0 macrophages cells had higher expression in low-risk score group, the expression of CD8 T cells, activated memory CD4 T cells and M1 macrophages cells were higher in the high-risk group. The infiltrating levels of CD8 T cells were associated with high-risk level and low OS (Jeong et al., 2017). The present and previous findings suggested that the infiltration of specific immune cells could cause bad prognosis.

It had some limitations in this work. First, the prognostic value of 14 IRGs was validated by two external data, but no *in vivo* or *in vitro* experimental study was carried out. Second, six IRGs (BACH2, FGF20, IFITM1, LMBR1L, MTCP1, and RFX5) part of risk model was all correlated to OS, but no difference was detected between low- and high-risk groups. The reason and rule of these IRGs were not explored.

In this work, we not only explored the role of the immune system in DLBCL development, but we provided an advanced treatment way.

## Conclusion and Outlook

In this work, a risk score formula was established based on the 14 IRGs signature in DLBCL. According to these signatures, our study might present valuable clinical applications in personalized and precise treatment. The result of GSEA enrichment analysis indicated that the deterioration of DLBCL involved natural killer cell-mediated cytotoxicity and other 11 pathways. These 12 pathways were valuable to further analyze in cell and animal testing. Two strong correlation (*R* > 0.8) were found between TNSF10 and TIM-3 (HAVCR2), LAP3 and TIM-3. The expression level of TNSF10 and LAP3 could provide basis and guidance for immunotherapy. the infiltration and potential immune checkpoint blockade immunotherapy could be predicted. Furthermore, 79 small molecular were screened as potential drugs for DLBCL. Nevertheless, our conclusion should be tested by other public data and verified in future research.

## Data Availability Statement

The original contributions presented in the study are included in the article/Supplementary Material, further inquiries can be directed to the corresponding author/s.

## Ethics Statement

Ethical review and approval was not required for the study on human participants in accordance with the local legislation and institutional requirements. Written informed consent for participation was not required for this study in accordance with the national legislation and the institutional requirements.

## Author Contributions

PF and GL were the major contributors in writing of this manuscript. GL was the responding author. YH, HL, and JP provided a few studies, ideas, and revised opinion. All authors have read and agreed to the published version of the manuscript.

## Conflict of Interest

The authors declare that the research was conducted in the absence of any commercial or financial relationships that could be construed as a potential conflict of interest.

## Footnotes

1 https://immport.niaid.nih.gov

2 http://sangerbox.com/AllTools?tool_id=9703341

3 http://gepia.cancer-pku.cn/

4 https://portals.broadinstitute.org/cmap

## Supplementary Material

Supplementary Figure 1 A flowchart for methods was shown.

Supplementary Figure 2 The proportion of immune cell infiltration between the low-risk group (red) and high-risk group (blue) (no significant differences between high and low risk groups).

## Abbreviations

DLBCL: diffuse large B cell lymphoma
NHL: non-hodgkin lymphoma
TCGA: the cancer genome atlas
GEO: gene expression omnibus
IRGs: immune-related genes
ImmPort: immunology database and analysis portal
OS: overall survival
LASSO: least absolute shrinkage and selection operator
K-M: Kaplan-meier
GSEA: gene set enrichment analysis
PD-L1: programmed cell death ligand 1
PD-1: programmed cell death protein 1
CTLA-4: cytotoxic T lymphocyte antigen-4
LAG-3: lymphocyte activation gene 3
TIM-3: T cell immunoglobulin-3.
