# Supplementary figures and images for: Identification of a 14-Gene Prognostic Signature for Diffuse Large B Cell Lymphoma (DLBCL)
Source: Front Genet. 2021 Feb 10;12:625414. doi: 10.3389/fgene.2021.625414 (PMC7902938; doi:10.3389/fgene.2021.625414)

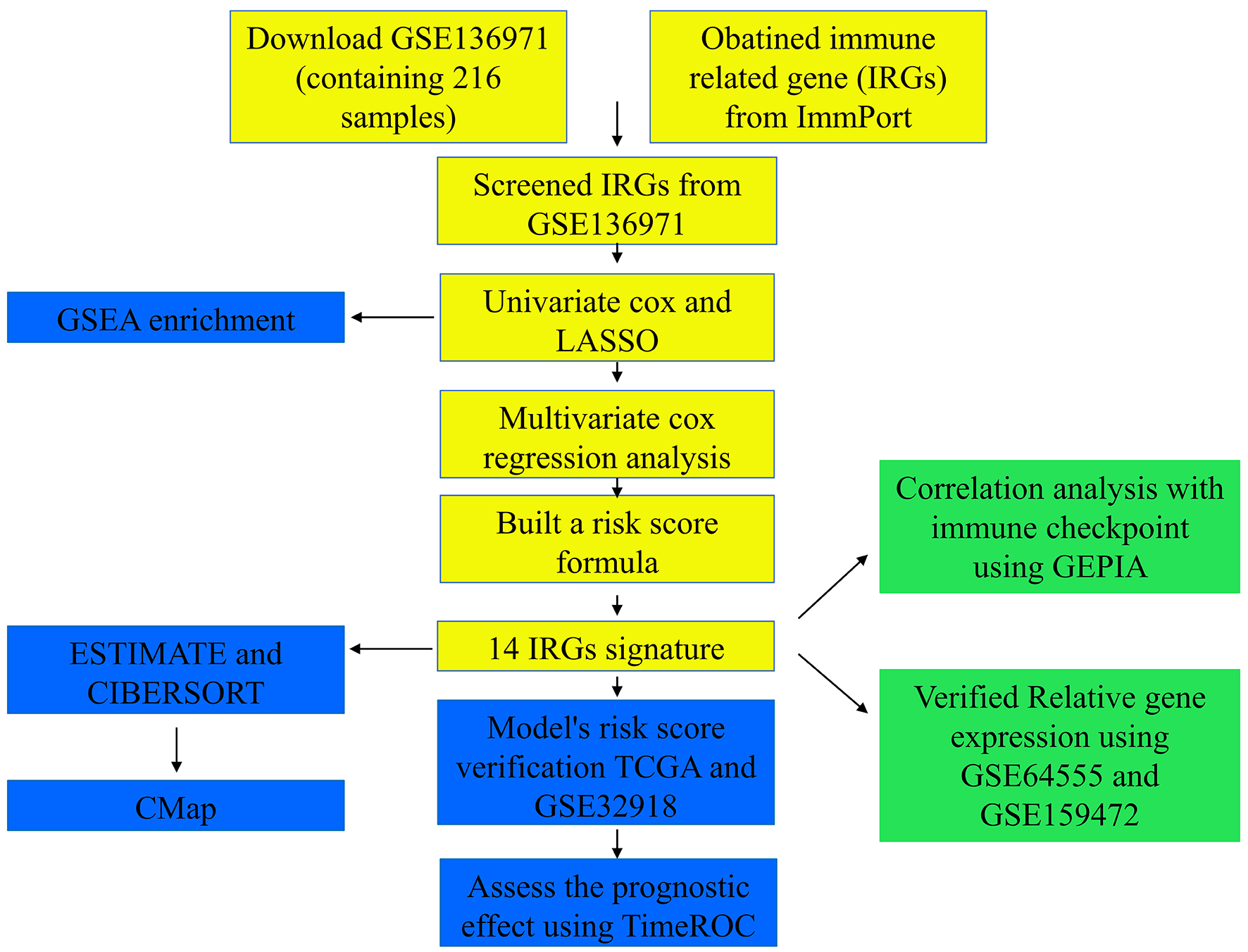

Supplement: Supplementary Figure 1 — A flowchart for methods was shown. [file Image_1.JPEG]

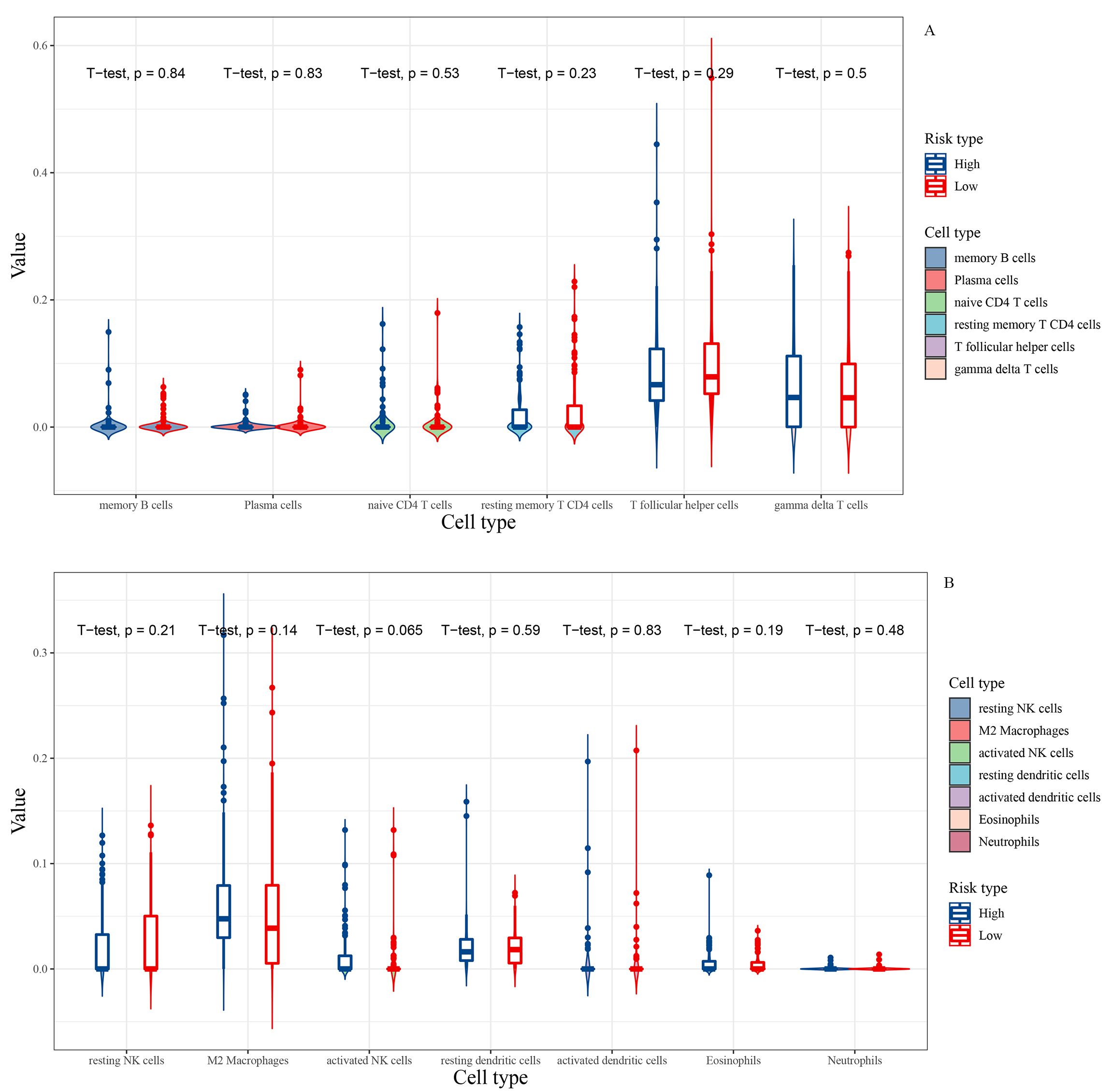

Supplement: Supplementary Figure 2 — The proportion of immune cell infiltration between the low-risk group (red) and high-risk group (blue) (no significant differences between high and low risk groups). [file Image_2.JPEG]
