# Supplementary material for: Identification of a 14-Gene Prognostic Signature for Diffuse Large B Cell Lymphoma (DLBCL)
Source: Front Genet. 2021 Feb 10;12:625414. doi: 10.3389/fgene.2021.625414 (PMC7902938; doi:10.3389/fgene.2021.625414)
Supplement: Supplementary file 4 [file Table_2.DOCX]

| rank | CMap name | Cell line | Mean CMap score | n | enrichment | p | specificity |
| --- | --- | --- | --- | --- | --- | --- | --- |
| 1 | trichostatin A | HL60 | -0.346 | 34 | -0.389 | 0.000 | 0.289 |
| 2 | vorinostat | HL60 | -0.688 | 3 | -0.979 | 0.000 | 0.011 |
| 3 | wortmannin | MCF7 | 0.306 | 10 | 0.682 | 0.000 | 0.128 |
| 4 | nefopam | MCF7 | -0.649 | 3 | -0.922 | 0.001 | 0.000 |
| 5 | mafenide | MCF7 | 0.762 | 2 | 0.976 | 0.001 | 0.000 |
| 6 | metoprolol | MCF7 | 0.770 | 2 | 0.971 | 0.001 | 0.011 |
| 7 | tiaprofenic acid | MCF7 | -0.671 | 2 | -0.970 | 0.002 | 0.000 |
| 8 | diethylstilbestrol | PC3 | 0.730 | 2 | 0.966 | 0.002 | 0.000 |
| 9 | loracarbef | MCF7 | 0.700 | 2 | 0.956 | 0.003 | 0.000 |
| 10 | (-)-isoprenaline | MCF7 | 0.680 | 2 | 0.956 | 0.003 | 0.000 |
| 11 | metformin | PC3 | 0.715 | 2 | 0.949 | 0.005 | 0.012 |
| 12 | foliosidine | PC3 | -0.716 | 2 | -0.949 | 0.006 | 0.000 |
| 13 | harmalol | MCF7 | 0.676 | 2 | 0.941 | 0.007 | 0.000 |
| 14 | orphenadrine | PC3 | -0.682 | 2 | -0.945 | 0.007 | 0.015 |
| 15 | citiolone | PC3 | -0.664 | 2 | -0.941 | 0.007 | 0.000 |
| 16 | noretynodrel | MCF7 | -0.612 | 2 | -0.941 | 0.008 | 0.021 |
| 17 | etoposide | MCF7 | 0.658 | 2 | 0.934 | 0.008 | 0.109 |
| 18 | BCB000038 | PC3 | -0.614 | 2 | -0.929 | 0.010 | 0.017 |
| 19 | LM-1685 | MCF7 | 0.535 | 3 | 0.826 | 0.011 | 0.000 |
| 20 | oxolamine | MCF7 | 0.627 | 2 | 0.926 | 0.011 | 0.006 |
| 21 | todralazine | MCF7 | 0.695 | 2 | 0.924 | 0.011 | 0.000 |
| 22 | valproic acid | HL60 | -0.319 | 14 | -0.411 | 0.012 | 0.362 |
| 23 | diprophylline | MCF7 | 0.705 | 2 | 0.922 | 0.012 | 0.007 |
| 24 | clofazimine | MCF7 | 0.652 | 3 | 0.813 | 0.013 | 0.041 |
| 25 | tanespimycin | HL60 | -0.342 | 12 | -0.436 | 0.013 | 0.386 |
| 26 | pancuronium bromide | MCF7 | -0.606 | 2 | -0.917 | 0.014 | 0.032 |
| 27 | gemfibrozil | MCF7 | 0.612 | 2 | 0.915 | 0.014 | 0.007 |
| 28 | mefloquine | MCF7 | 0.616 | 2 | 0.913 | 0.015 | 0.080 |
| 29 | mepenzolate bromide | MCF7 | -0.569 | 2 | -0.909 | 0.017 | 0.017 |
| 30 | telenzepine | MCF7 | 0.656 | 2 | 0.908 | 0.017 | 0.007 |
| 31 | demecarium bromide | MCF7 | -0.581 | 2 | -0.907 | 0.017 | 0.083 |
| 32 | isoniazid | PC3 | 0.697 | 2 | 0.908 | 0.017 | 0.046 |
| 33 | memantine | MCF7 | -0.568 | 2 | -0.905 | 0.018 | 0.007 |
| 34 | 0317956-0000 | PC3 | -0.379 | 4 | -0.695 | 0.018 | 0.071 |
| 35 | amoxicillin | MCF7 | 0.610 | 2 | 0.903 | 0.019 | 0.019 |
| 36 | 15(S)-15-methylprostaglandin E2 | MCF7 | 0.599 | 2 | 0.902 | 0.020 | 0.018 |
| 37 | puromycin | MCF7 | 0.636 | 2 | 0.899 | 0.021 | 0.204 |
| 38 | thioproperazine | PC3 | -0.542 | 2 | -0.896 | 0.022 | 0.013 |
| 39 | iproniazid | PC3 | 0.642 | 2 | 0.892 | 0.024 | 0.028 |
| 40 | geldanamycin | PC3 | -0.535 | 2 | -0.889 | 0.024 | 0.059 |
| 41 | pyrvinium | MCF7 | 0.478 | 4 | 0.675 | 0.025 | 0.305 |
| 42 | altizide | MCF7 | 0.601 | 2 | 0.888 | 0.026 | 0.024 |
| 43 | monastrol | MCF7 | 0.363 | 7 | 0.518 | 0.027 | 0.077 |
| 44 | amiloride | MCF7 | 0.608 | 2 | 0.883 | 0.028 | 0.009 |
| 45 | amiodarone | MCF7 | 0.311 | 3 | 0.757 | 0.028 | 0.082 |
| 46 | propidium iodide | MCF7 | 0.565 | 2 | 0.878 | 0.030 | 0.000 |
| 47 | disulfiram | MCF7 | -0.565 | 2 | -0.877 | 0.030 | 0.118 |
| 48 | amoxapine | MCF7 | 0.577 | 2 | 0.877 | 0.031 | 0.041 |
| 49 | 15(S)-15-methylprostaglandin E2 | PC3 | 0.583 | 2 | 0.876 | 0.031 | 0.095 |
| 50 | sirolimus | PC3 | 0.288 | 8 | 0.481 | 0.031 | 0.400 |
| 51 | antazoline | MCF7 | 0.561 | 2 | 0.875 | 0.031 | 0.013 |
| 52 | acebutolol | PC3 | -0.539 | 2 | -0.873 | 0.032 | 0.033 |
| 53 | cefalotin | MCF7 | -0.529 | 2 | -0.872 | 0.032 | 0.010 |
| 54 | epitiostanol | MCF7 | -0.548 | 2 | -0.871 | 0.033 | 0.122 |
| 55 | 0316684-0000 | MCF7 | 0.590 | 2 | 0.872 | 0.033 | 0.035 |
| 56 | colforsin | PC3 | 0.544 | 2 | 0.870 | 0.035 | 0.129 |
| 57 | raloxifene | MCF7 | 0.484 | 3 | 0.733 | 0.037 | 0.102 |
| 58 | metoclopramide | MCF7 | -0.440 | 3 | -0.737 | 0.037 | 0.012 |
| 59 | lovastatin | MCF7 | -0.576 | 2 | -0.863 | 0.038 | 0.065 |
| 60 | esculetin | MCF7 | -0.599 | 2 | -0.857 | 0.041 | 0.060 |
| 61 | dexibuprofen | MCF7 | 0.556 | 2 | 0.857 | 0.041 | 0.021 |
| 62 | ascorbic acid | MCF7 | 0.652 | 2 | 0.857 | 0.042 | 0.065 |
| 63 | diflunisal | MCF7 | 0.545 | 2 | 0.856 | 0.042 | 0.000 |
| 64 | bumetanide | MCF7 | 0.571 | 2 | 0.856 | 0.042 | 0.129 |
| 65 | sotalol | MCF7 | -0.503 | 2 | -0.853 | 0.043 | 0.011 |
| 66 | sulfadoxine | MCF7 | -0.528 | 2 | -0.853 | 0.043 | 0.048 |
| 67 | benzamil | MCF7 | 0.324 | 3 | 0.720 | 0.044 | 0.064 |
| 68 | diphemanil metilsulfate | MCF7 | 0.630 | 2 | 0.851 | 0.044 | 0.036 |
| 69 | gibberellic acid | MCF7 | -0.503 | 2 | -0.850 | 0.045 | 0.106 |
| 70 | methyldopate | MCF7 | -0.505 | 2 | -0.849 | 0.045 | 0.200 |
| 71 | isoniazid | MCF7 | 0.528 | 2 | 0.847 | 0.047 | 0.006 |
| 72 | nifenazone | PC3 | 0.604 | 2 | 0.847 | 0.047 | 0.059 |
| 73 | dihydrostreptomycin | MCF7 | 0.530 | 2 | 0.846 | 0.048 | 0.056 |
| 74 | dapsone | MCF7 | 0.613 | 2 | 0.845 | 0.048 | 0.070 |
| 75 | 16-phenyltetranorprostaglandin E2 | MCF7 | -0.517 | 2 | -0.844 | 0.049 | 0.053 |
| 76 | oleandomycin | MCF7 | -0.507 | 2 | -0.844 | 0.049 | 0.028 |
| 77 | metampicillin | PC3 | 0.642 | 2 | 0.844 | 0.049 | 0.097 |
| 78 | pirinixic acid | MCF7 | 0.559 | 2 | 0.843 | 0.050 | 0.016 |
| 79 | ethosuximide | PC3 | -0.521 | 2 | -0.842 | 0.050 | 0.023 |
| 80 | aztreonam | MCF7 | 0.549 | 2 | 0.841 | 0.051 | 0.075 |
